# Maximal fat oxidation assessment in active postmenopausal females: A novel relative power FATmax test

**DOI:** 10.14814/phy2.70991

**Published:** 2026-06-30

**Authors:** Jordi Monferrer‐Marín, Ainoa Roldán, Jørn Wulff Helge, Cristina Blasco‐Lafarga

**Affiliations:** ^1^ Sport Performance and Physical Fitness Research Group (UIRFIDE), Physical Education and Sports Department University of Valencia Valencia Spain; ^2^ Department of Biomedical Sciences, Faculty of Health and Medical Sciences University of Copenhagen Copenhagen Denmark

**Keywords:** aging, energy expenditure, metabolic flexibility, relative power, respiratory exchange

## Abstract

This study compared a novel relative‐to‐body‐mass FATmax test (RFT; 0.15 W/kg/4 min from 0.45 W/kg) with a traditional absolute power FATmax test (AFT; 10 W/3min15sec from 30 W) in postmenopausal females. The aim was to determine whether the RFT protocol would improve fat oxidation kinetics during exercise, in females with lower maximal fat oxidation (MFO). It was hypothesized that the longer duration and the lower workload increments would result in higher fat oxidation values than AFT. Seventeen active postmenopausal females (69.2 ± 5.1 years) performed both protocols in a randomized order and were divided into above (H‐MFO) or below (L‐MFO) 0.3 g/min MFO. Groups were equal in age and body composition. Overall, time to MFO was delayed during the RFT protocol (765 ± 621 vs. 401 ± 262 s) without MFO differences. Both groups displayed longer time to MFO in RFT, whilst significantly only for L‐MFO (420 ± 280 s vs. 317 ± 232). Although no statistical differences, moderate effect sizes were observed in the L‐MFO group during RFT for both MFO (0.22 ± 0.04 vs. 0.19 ± 0.06 g/min, *d* = 0.65) and VO_2_ (12.8 ± 3.8 vs. 15.4 ± 5.5 mL/min/kg, *d* = −0.53). RFT may improve the determination of substrate oxidation kinetics in postmenopausal females with reduced fat oxidation capacity, while facilitating complementary analyses requiring longer recordings and stability (efficiency and Heart Rate Variability).

## 1 INTRODUCTION

Metabolic flexibility (MF) is the capacity to adapt and adjust substrate oxidation relative to substrate availability (Broskey et al., 2020; Kelley et al., 1999). Aging is associated with reduced fat oxidation rates (Dahlgaard Hansen et al., 2024), related to a decline in mitochondrial content (St‐Jean‐Pelletier et al., 2017) and mitochondrial respiratory capacity (Hepple, 2016). In parallel, age‐associated declines also lead to an impaired ability to increase carbohydrate oxidation when required (Reynolds et al., 2019), both affecting metabolic flexibility.

Regarding the menopausal transition, this is characterized by a marked decline in ovarian hormones, particularly 17β‐estradiol and progesterone, together with alterations in androgen availability and increased circulating concentrations of gonadotropins, including follicle‐stimulating hormone (FSH) and luteinizing hormone (LH), reflecting the progressive loss of ovarian negative feedback on the hypothalamic–pituitary axis (Burger, 2008). These endocrine changes have been associated with alterations in substrate utilization (Abildgaard et al., 2013), increased visceral adiposity and reductions in muscle mass and strength, which together influence energy expenditure (Kang et al., 2023; Vieira‐Potter et al., 2026) and metabolic flexibility (Blasco‐Lafarga et al., 2022; Kleis‐Olsen et al., 2024) beyond the age‐associated impairments. However, despite its physiological relevance, metabolic flexibility in postmenopausal females remains poorly characterized, particularly during exercise.

Recent evidence suggests that metabolic flexibility in this population may depend more on muscle function than on chronological aging per se (Monferrer‐Marín et al., 2022; Russ et al., 2025). In this context, FATmax test are incremental exercise protocols designed to identify maximal fat oxidation (MFO) and the exercise intensity at which it occurs (FATmax), typically through indirect calorimetry and progressive workload increments (Maunder et al., 2018). However, most FATmax protocols have been developed and validated in younger or mixed populations, with considerable methodological variability in exercise modality, warm‐up procedures, stage duration, incremental workload and nutritional status (Amaro‐Gahete et al., 2019; Maunder et al., 2018). This methodological variability may be particularly relevant in postmenopausal females. Chrzanowski‐Smith et al. (2018) reported delayed steady‐state attainment in this population, so the commonly used stage durations (e.g., 3 min) may be insufficient to accurately capture their substrate oxidation kinetics. In addition, the high interindividual variability in fat oxidation rates (Blasco‐Lafarga et al., 2022) and body composition parameters may limit the suitability of absolute workloads prescriptions.

Previous studies have applied FATmax protocols using different exercise modalities and workload progressions. Tan et al. (2018) used a treadmill FATmax test comprised of 3‐min stages with 0.5 km/h increments over the initial 3.5 km/h, until reaching a RER >1.0. Recent cycle‐ergometer studies have also used 3‐min stages but varying starting workloads and increments adjusted according to participants fitness levels (Dahlgaard Hansen et al., 2024; Kleis‐Olsen et al., 2024). In postmenopausal females, Monferrer‐Marín et al. (2022) used an intermediate protocol (starting at 30 W with increments of 10 W, 3‐min 15 s stages), reporting low energy expenditure and a downward and leftward shift in substrate oxidation rate curves compared to younger populations. These findings suggest that protocols originally developed in younger adults may not be fully appropriate for postmenopausal females (Monferrer‐Marín et al., 2022), as fixed absolute workloads may impose a disproportionately higher relative metabolic demand, potentially leading to an earlier shift toward carbohydrate reliance. As a result, this may compromise the accurate characterization of substrate oxidation evolution. Taken together, these limitations highlight the need for FATmax protocols tailored to the physiological characteristics of postmenopausal females to improve the accuracy of MFO and FATmax determination, mostly in those with lower fat oxidation rates.

Therefore, the aim of this study was to compare a new Relative to body mass FATmax test (RFT; starting at 0.45 W/kg and increasing by 0.15 W/kg of body mass every 4 min), with a traditional Absolute Power FATmax test (AFT; starting at 30 W and increasing by 10 W every 3 min 15 s) in postmenopausal females. By scaling workload relative to body mass and extending stage duration, the RFT was designed to attain better steady state and thus optimize substrate oxidation kinetics during exercise. Accordingly, we hypothesize that the RFT will allow postmenopausal females to achieve higher fat oxidation compared with the AFT.

## 2 MATERIALS & METHODS

### 2.1 Ethical approval

The study registered at ClinicalTrials.gov (NCT06336070). All participants volunteered to participate and provided written informed consent before the initiation of any study procedures or data collection. The protocol was approved by the Science Ethics Committee (2024‐FIS‐3251696) and conducted in accordance with the ethical standards set forth in the Declaration of Helsinki.

### 2.2 Study objectives

The primary objective was to compare two FATmax test protocols in postmenopausal females to determine whether a relative power protocol (RFT) would elicit higher maximal fat oxidation due to better steady‐state substrate oxidation kinetics compared with a traditional absolute power protocol (AFT). To further explore whether protocols differed according to oxidative capacity, the sample was divided into two groups based on their MFO values: a Low MFO group (L‐MFO) exhibiting a MFO value below the median FATox value of 0.3 g/min, and a High MFO group (H‐MFO) exhibiting values above this threshold.

The cut‐off point of 0.3 g/min was selected based on reference data (50th percentile) from sedentary middle‐aged adults (Amaro‐Gahete et al., 2018), and recent data in postmenopausal females that report mean MFO values of 0.29 g/min (Kleis‐Olsen et al., 2024). Given that the present cohort consisted of physically active postmenopausal females, this threshold was considered a physiologically meaningful and conservative criterion to differentiate lower and higher fat oxidation capacity.

### 2.3 Participants

Eighteen older females were recruited to participate in the study. An a priori sample size calculation was conducted using G*Power (version 3.1.9.6; Heinrich‐Heine‐Universität Düsseldorf, Germany), based on the primary outcome of MFO. The calculation assumed an expected within‐subject difference of approximately 0.05 g/min in MFO between protocols, with an estimated standard deviation of 0.07 g/min, based on variability reported in previous studies in adult populations (Amaro‐Gahete et al., 2018). These assumptions corresponded to an expected effect size of Cohen's dz. = 0.71. Using a two‐tailed paired‐samples *t*‐test (difference between two dependent means), with an alpha level of 0.05, and a desired statistical power of 0.80, this analysis indicated a minimum of 18 participants. Seventeen participants completed the study, corresponding to an achieved statistical power of 0.79.

The inclusion criteria were as follows: (1) females over 60 years, (2) moderately active according to the International Physical Activity Questionnaire (IPAQ, >600 METs/week), (3) >12 consecutive months of amenorrhoea, and (4) no medical contraindications for physical exercise. The exclusion criteria included: (1) diagnosed with any chronic disease, (2) use of medications (e.g., beta‐blockers) that limit or affect physical activity, (3) undergoing hormone replacement therapy or any estrogen treatment, and (4) episodes of hypotensive response to exercise.

One participant was excluded due to a hypotensive response to the first incremental protocol and her participation was discontinued on the third day of evaluations (second test), therefore she did not complete the study.

### 2.4 Study outline

**FIGURE 1:**
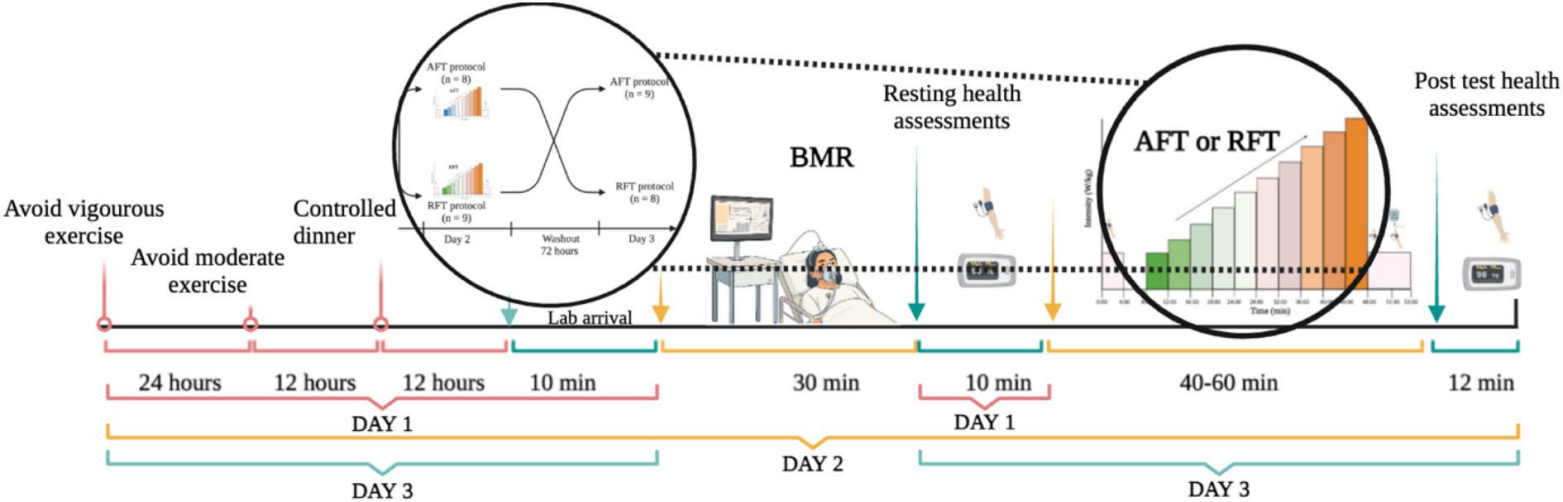
Experimental procedure. BMR, basal metabolic rate; AFT, absolute power FATmax test; RFT, relative‐to‐body‐mass FATmax test.

Overall, 17 active postmenopausal female participants (69.2 ± 5.1 yrs.; 41.3 ± 5.2 fat‐free mass kg) completed the 3‐day study with both tests (AFT & RFT) performed in randomized order and with at least 48 h of rest between each protocol. On the first day, baseline measurements were taken, including blood pressure (BP), oxygen saturation (SaO_2_), and anthropometric assessment, and participants were familiarized with the cycle ergometer given their irregular use of cycling or lack of familiarity with the cycle ergometer. On the remaining two other days, the measurements were repeated, except for the recording of basal metabolic rate, which was performed on day 2 for all participants (Figure 1). Nutrition was standardized, ensuring a minimum carbohydrate intake of total energy intake of 550 kJ of the total 900 kJ at the previous dinner. In addition, they were asked to replicate the same meal before each testing session to minimize intraindividual variability. All participants reported to the laboratory following a 12 h overnight fast, and tests were conducted at the same time (either 8:30 am or 11:00 am). Additionally, participants were asked to avoid moderate and intense physical exercise for 24 h, and 48 h before, respectively, both the second and third days.

### 2.5 Experimental methods

Participants reported to the laboratory and resting heart rate (HR) was measured for 10 min using a Polar H10 band (Polar Electro Oy, Kempele, Finland). Subsequently, arterial oxygen saturation (SaO2) was recorded using the Nonin Onyx Vantage 9590 Finger‐Pulse Oximeter (Nonin Medical, Plymouth, Minnesota, USA), and blood pressure (BP) was measured three times before, as well as after the test, with two posttest measurements, one during the recovery period on the bike, as well as at the end of the 5 min recovery period. The BP measurements were done with the Omron M6 sphygmomanometer (HEM‐7420, Omron Healthcare, Kyoto, Japan).

A brief interview was then conducted to assess the health status of the participants and their level of physical activity using the IPAQ questionnaire. Height was measured (SECA 222, Hamburg, Germany) and body composition was determined by bioelectrical impedance (Tanita DC‐430 MA S; Tokyo, Japan). Muscle power, both relative and allometric, was evaluated using the 5‐repetition sit‐to‐stand test (5STS) as described by Alcazar et al. (2018). Finally, a familiarization session with the cycle ergometer and the Saris H3 smart trainer (CycleOps Hammer Direct Drive Trainer, Saris, Madison, USA) was conducted, where cadence and biomechanical adjustments were set for the participant's comfort during the incremental test. On the second day, an evaluation of the basal metabolic rate (BMR) by indirect calorimetry using a K5 metabolic cart (Cosmed, Rome, Italy) was conducted (Fullmer et al., 2015).

Before the warm‐up on both test days SaO_2_ and BP measurements were repeated, and a baseline lactate measurement was taken using the Lactate Scout Sport analyzer (SensLab GmbH, Leipzig, Germany). The warm‐up was performed with the same duration and intensity as the first stage in the testing protocol (i.e., 3′15″ in AFT at 30 W, or 4′ at 0.45 W/kg in RFT). As previously detailed, the 30 W AFT (Monferrer‐Marín et al., 2022) starts at 30 W and increases by 10 W every 3 min 15 s for all participants equally. Conversely, the RFT starts at 0.45 W/kg and increases by 0.15 W/kg every 4 min, so the power output intensities are individually adjusted for each participant.

The incremental test ended when at least two of the following criteria were met: (1) RER exceeding 1, (2) rate of perceived exertion (RPE) exceeding 6 on the Borg 0–10 scale, (3) visual analog scale of pain (VAS) >6, or (4) SaO_2_ below 92%.

VO_2_ and VCO_2_ were measured by indirect calorimetry using the K5 metabolic card (Cosmed, Rome, Italy). The online gas analyzers were carefully calibrated with an automated volume calibration and with a gas mixture recommended by the manufacturer prior to the start of each test. The last 60 s in each intensity were then applied to calculate whole‐body fat oxidation rates (Amaro‐Gahete et al., 2019), where the substrate oxidation was calculated using Frayn's equation, with the assumption that the urinary nitrogen excretion rate was negligible (Frayn, 1983):

(1) $$\text{FATox}\;\left(g/\min\right):1.67{\mathrm{VO}}_{2}\;\left(l/\min\right)–1.67{\mathrm{VCO}}_{2}\;\left(l/\min\right)$$

(2) $$\text{CHOox}\;\left(g/\min\right):4.55{\mathrm{VCO}}_{2}\;\left(l/\min\right)–3.21{\mathrm{VO}}_{2}\;\left(l/\min\right)$$

The FATox value at MFO was calculated using the FFM value (mg/min/kg FFM), as it may be more appropriate when making comparisons in a heterogeneous sample (Waldman et al., 2023). The median of the absolute value (0.3 g/min) was used to divide the groups into low MFO (L‐MFO) for those females below this value and high MFO (H‐MFO) for females above.

SaO_2_ was monitored throughout the protocol alongside the HR. Similarly, RPE along with the VAS, for pain (Hicks et al., 2001), was recorded. Both scales range from 0 to 10, where 10 indicates maximum exertion or pain, respectively. Additionally, both blood pressure and lactate levels were measured posttest, and after 1 and 5 min for blood pressure, and at 3 min for lactate.

### 2.6 Statistical analysis

The statistical analyses and figures were performed using RStudio 4.0.2 (R Core Team, Vienna, Austria). After testing for normality (Shapiro–Wilk test), descriptive statistics, including mean ± standard deviation (SD) were calculated for the sample characteristics and for the intensities and key variables describing the test, in addition to the coefficient of variation (CV%) of VO_2_ (Tables 1, 2, 3). Mann–Whitney *U* and *T*‐tests were performed to compare the groups (L‐MFO vs. H‐MFO) and protocols (RFT vs. AFT), and a Repeated Measures ANOVA was used to compare the intensities evolution between protocols and steps in heart rate and RER, and Friedman analysis was used for relative power. Effect sizes were calculated using Cohen's *d* for pairwise comparisons. Further violin plots were generated using the “ggplot2”, “dplyr” and “patchwork” packages in R studio comparing AFT and RFT protocols for both groups in RER and FATox values during the first four steps of the test. Bland–Altman plots were additionally constructed with 95% limits of agreement (LoA) (Bland & Altman, 1986), to compare both protocols for MFO, including the calculation of mean bias and assessment of the coefficient of determination (*R* ^2^). These analyses provide insights into the distribution, central tendency, reliability, and agreement of the performance measures obtained from AFT and RFT. The significance level was set at *p* < 0.05 for all tests.

## 3 RESULTS

The main descriptive data show the groups were very similar (Table 1), despite the difference in MFO that was used to allocate the groups. No significant differences were observed between groups for body weight (*p* = 0.25) or fat‐free mass (*p* = 0.32), although moderate effect sizes were noted, likely influenced by the relatively small sample size per group. Resting systolic blood pressure values were elevated in both groups (135 ± 14 vs. 128 ± 19 mmHg), whereas diastolic blood pressure remained within normal ranges (79.5 ± 6.8 vs. 78.0 ± 6.2 mmHg), with no significant between‐group differences.

**TABLE 1:** Comparative descriptive data for females with MFO above or below 0.3 g/min.

Variable	L‐MFO (*n* = 8)	H‐MFO (*n* = 9)	Effect size
Age (yrs.)	68.9 ± 4.7	69.0 ± 5.9	−0.02
Weight (kg)	58.2 ± 10.2	65.5 ± 14.5	−0.58
Fat mass (% body mass)	31.0 ± 5.7	34.0 ± 6.7	−0.48
Fat‐free mass (kg)	39.8 ± 5.0	42.4 ± 5.5	−0.50
SBP (mmHg)	135 ± 14	128 ± 19	0.41
DBP (mmHg)	79.5 ± 6.8	78.0 ± 6.2	0.23
HR (bpm)	70.7 ± 4.2	65.2 ± 3.3	0.12
Relative power 5STS test (W/kg)	3.3 ± 0.7	3.3 ± 0.5	−0.15
Allometric power 5STS test (W/m^2^)	78.4 ± 23.5	84.0 ± 78.5	−0.32

*Note*: Data are presented as mean ± standard deviation.

Abbreviations: 5STS, five times sit‐to‐stand test; DBP, diastolic blood pressure; H‐MFO, high MFO group; HR, heart rate; L‐MFO, low MFO group; SBP, systolic blood pressure; yrs., years.

### 3.1 Comparison of physiological responses between FATmax protocols

As reported in Table 2, no differences between protocols were observed at MFO intensity for absolute FATox (*p* = 0.75), FATox relative to fat‐free mass (*p* = 0.73), relative power output (*p* = 0.9), oxygen consumption (*p* = 0.79), rating of perceived exertion (*p* = 0.57), or heart rate (*p* = 0.72). However, the time before reaching the FATmax was significantly longer in RFT (*p* < 0.05), with a medium‐large effect size (Cohen's *d* = −0.51). At the intensity corresponding to maximal carbohydrate oxidation (MCO), no differences between protocols were observed in relative power output, oxygen consumption, RPE, or HR. However, time to MCO (CHOmax) was significantly longer in the RFT (*p* < 0.001), with a large effect size (Cohen's *d* = −1.04).

**TABLE 2:** Protocol data at intensities spectrum.

	AFT	RFT	Effect size
MFO intensity			
MFO (g/min)	0.30 ± 0.12	0.28 ± 0.09	0.11
MFO (mg/min/kg FFM)	7.2 ± 3.0	7.0 ± 2.3	0.12
RelPower (W/kg)	0.74 ± 0.27	0.74 ± 0.32	−0.01
Time to FATmax (sec)	401 ± 262	765 ± 621*	−0.51
VO_2_ (mL/min/kg)	15.9 ± 5.3	15.5 ± 5.2	0.09
RPE	2.1 ± 2.0	2.5 ± 2.1	−0.20
HR (bpm)	101.9 ± 14.4	103.8 ± 15.0	−0.13
MCO intensity			
RelPower (W/kg)	1.21 ± 0.36	1.13 ± 0.26	0.24
Time to CHOmax (sec)	1170 ± 258	1553 ± 258**	−1.04
VO_2_ (mL/min/kg)	23.0 ± 5.8	22.8 ± 4.6	0.05
RPE	6.6 ± 1.6	6.4 ± 1.8	0.10
HR (bpm)	135.5 ± 13.7	131.5 ± 12.4	0.30
Recovery (3 min)			
bLa (mmol/L)	5.98 ± 1.91	5.45 ± 2.37	0.25
HR (bpm)	123.1 ± 12.9	113.3 ± 12.6*	0.75

*Note*: Data are presented as mean ± standard deviation. *p < 0.05, **p < 0.001.

Abbreviations: AFT, absolute FATmax test; bLa, blood lactate; CHOmax, intensity at which MCO was reached; FATmax, intensity at which MFO was reached; HR, heart rate; MCO, maximal carbohydrate oxidation rate; MFO, maximal fat oxidation rate; RFT, relative to body mass kg FATmax test; SaO_2_, arterial oxygen saturation; VO_2_, oxygen consumption.

During recovery (3 min posttest), blood lactate concentrations did not differ between protocols (Table 2). In contrast, heart rate was significantly lower following the RFT compared with the AFT (*p* < 0.05), with a moderate‐to‐large effect size (Cohen's *d* = 0.75).

Table 3 outlines the four first intensities in the test (RER <0.9) from a mechanical (relative power), metabolic (RER), and cardiovascular perspective (HR). Significant differences were observed between steps; notwithstanding, only relative power showed differences between protocols, with larger relative power in the AFT in steps 1 and 4.

**TABLE 3:** Protocol comparisons along the fat‐predominant intensities (steps 1 to 4).

Step	Relative power (W/kg)		RER		HR (bpm)	
	AFT	RFT	AFT	RFT	AFT	RFT
1	0.59 ± 0.11*	0.52 ± 0.07	0.79 ± 0.06	0.78 ± 0.53	89.2 ± 14.0	89.1 ± 11.6
2	0.71 ± 0.15###	0.64 ± 0.08###	0.84 ± 0.07###	0.83 ± 0.06###	97.6 ± 12.7##	97.1 ± 12.5#
3	0.84 ± 0.16###	0.75 ± 0.06###	0.87 ± 0.08###	0.86 ± 0.06#	106 ± 15.4###	105 ± 11.7##
4	0.98 ± 0.18*###	0.87 ± 0.06###	0.90 ± 0.09#	0.88 ± 0.07	113 ± 20.3##	112 ± 13.1##

*Note*: Data are presented as mean ± standard deviation. #*p* < 0.05, ##*p* < 0.01, ###*p* < 0.001 for Repeated Measures differences between intensities, *p < 0.05 for intergroup differences.

Abbreviations: AFT, absolute FATmax test; HR, heart rate; RER, respiratory exchange ratio; RFT, relative to body mass kg FATmax test.

Regarding the intergroup analysis, Table 4 outlines significant differences for absolute and relative to fat‐free mass FATox in both protocols. In the intragroup protocol comparison, RFT showed longer duration to MFO both in the L‐MFO group and in the H‐MFO group, with moderate effect size in both (Cohen's *d* = −0.59; −0.67). Despite some other effect sizes, no differences between protocols were observed in relative power output, VO_2_, RPE, or HR at MFO intensity in either group. Similarly, at MCO intensity, no protocol differences were observed in relative power output, VO_2_, RPE, or HR in either group. However, the time required to reach CHOmax was significantly longer in the RFT in both the L‐MFO and H‐MFO groups (*p* < 0.05), with large effect sizes (Cohen's *d* = −1.69 and − 1.01, respectively). Blood lactate concentrations during recovery did not differ between protocols in either group.

**TABLE 4:** Intergroup analysis and protocol comparison.

Variable	L‐MFO			H‐MFO		
	AFT	RFT	Effect size	AFT	RFT	Effect size
MFO intensity						
MFO (g/min)	0.19 ± 0.06	0.22 ± 0.04	−0.65	0.39 ± 0.06*	0.34 ± 0.07*	0.79
MFO (mg/min/kgFFM)	4.15 ± 1.41	4.84 ± 0.96	−0.57	9.44 ± 2.07*	8.23 ± 2.32*	0.34
RelPower (W/kg)	0.71 ± 0.30	0.60 ± 0.17	0.56	0.76 ± 0.30	0.86 ± 0.38	−0.31
Time to FATmax (sec)	317 ± 232	420 ± 280^#^	−0.59	444 ± 265	1010 ± 690	−0.67
VO_2_ (mL/min/kg)	15.4 ± 5.5	12.8 ± 3.8	0.53	16.5 ± 5.4	17.2 ± 5.4	−0.25
RPE	2.6 ± 1.6	2.3 ± 1.9	0.18	1.6 ± 1.7	2.7 ± 1.7	−0.54
HR (bpm)	102.7 ± 17.3	100.5 ± 9.7	0.10	101.3 ± 17.3	106.9 ± 17.3	−0.41
MCO intensity						
RelPower (W/kg)	1.08 ± 0.14	1.03 ± 0.10	0.44	1.32 ± 0.45	1.19 ± 0.45	0.24
Time to CHOmax (sec)	999 ± 193	1350 ± 220^#^	−1.69	1322 ± 213	1733 ± 533^#^	−1.01
VO_2_ (mL/min/kg)	21.3 ± 4.7	21.3 ± 3.9	0.00	24.6 ± 6.6	23.2 ± 6.6	0.09
RPE	7.0 ± 1.2	6.9 ± 1.9	0.11	7.3 ± 1.2	7.1 ± 3.0	0.09
HR (bpm)	133.4 ± 12.5	129.1 ± 15.3	0.38	137.1 ± 18.8	130.7 ± 13.7	0.42
Recovery						
bLa (mmol/L)	6.25 ± 2.16	6.55 ± 1.22	−0.17	5.74 ± 1.76	4.62 ± 1.76	0.55

*Note*: Data are presented as mean ± standard deviation. Intergroup differences: *p < 0.05; Intragroup protocol differences: #*p* < 0.05.

Abbreviations: bLa, blood lactate; CHOmax, intensity at which MCO was reached; FATmax, intensity at which MFO was reached; H‐MFO, high MFO group; HR, heart rate; kgFFM, fat‐free mass; L‐MFO, low MFO group; MCO, maximal carbohydrate oxidation rate; MFO, maximal fat oxidation rate; RelPower, relative power; VO_2_, oxygen consumption.

**FIGURE 2:**
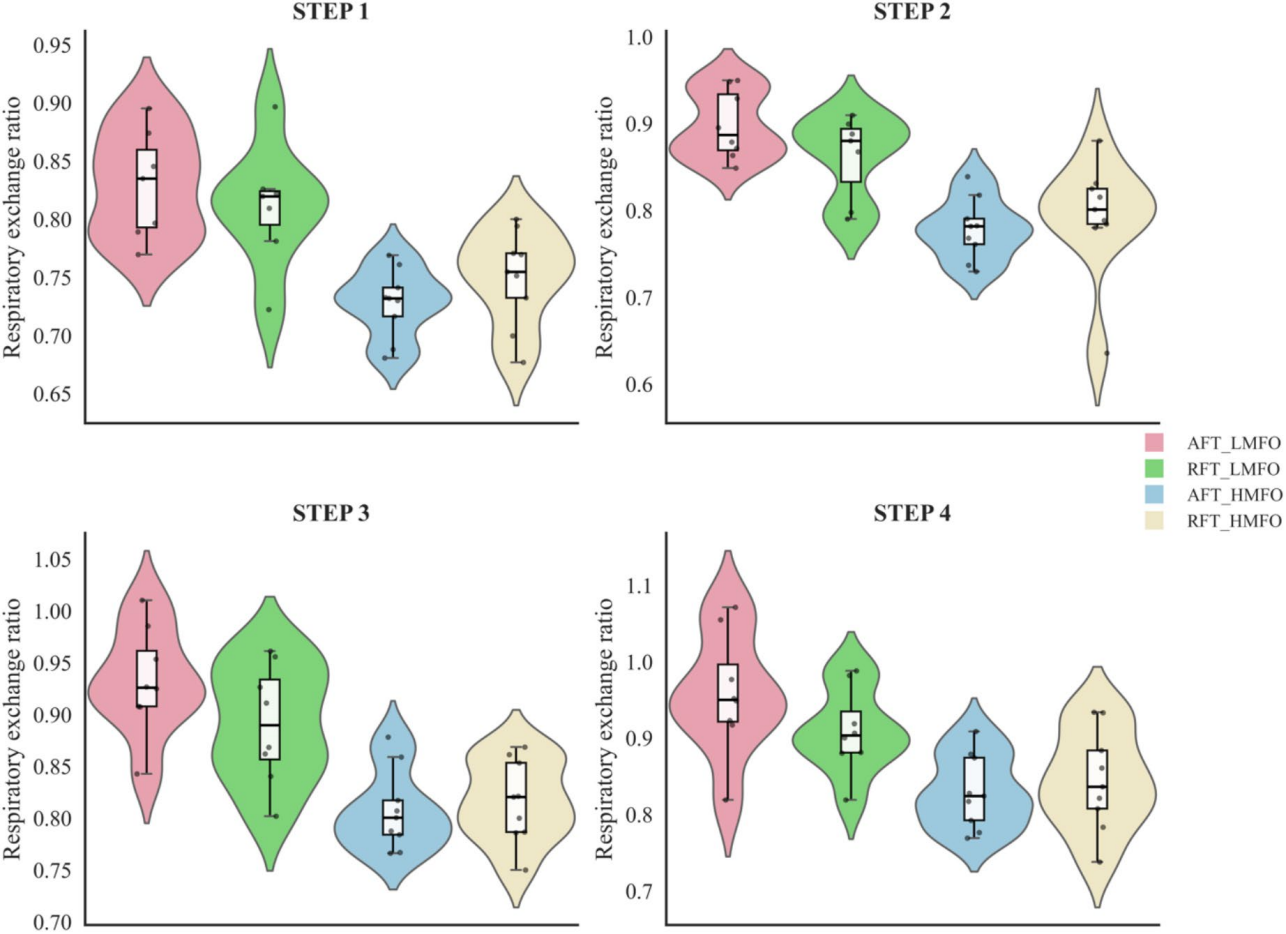
Violin plot of respiratory exchange ratio distribution around the first four steps of each test: Absolute FATmax test (AFT) versus Relative to body mass FATmax test (RFT). The internal box plot represents the median and interquartile range, while whiskers indicate the data range. MFO, Maximal Fat Oxidation Point; L‐MFO, low MFO group; H‐MFO, high MFO group.

**FIGURE 3:**
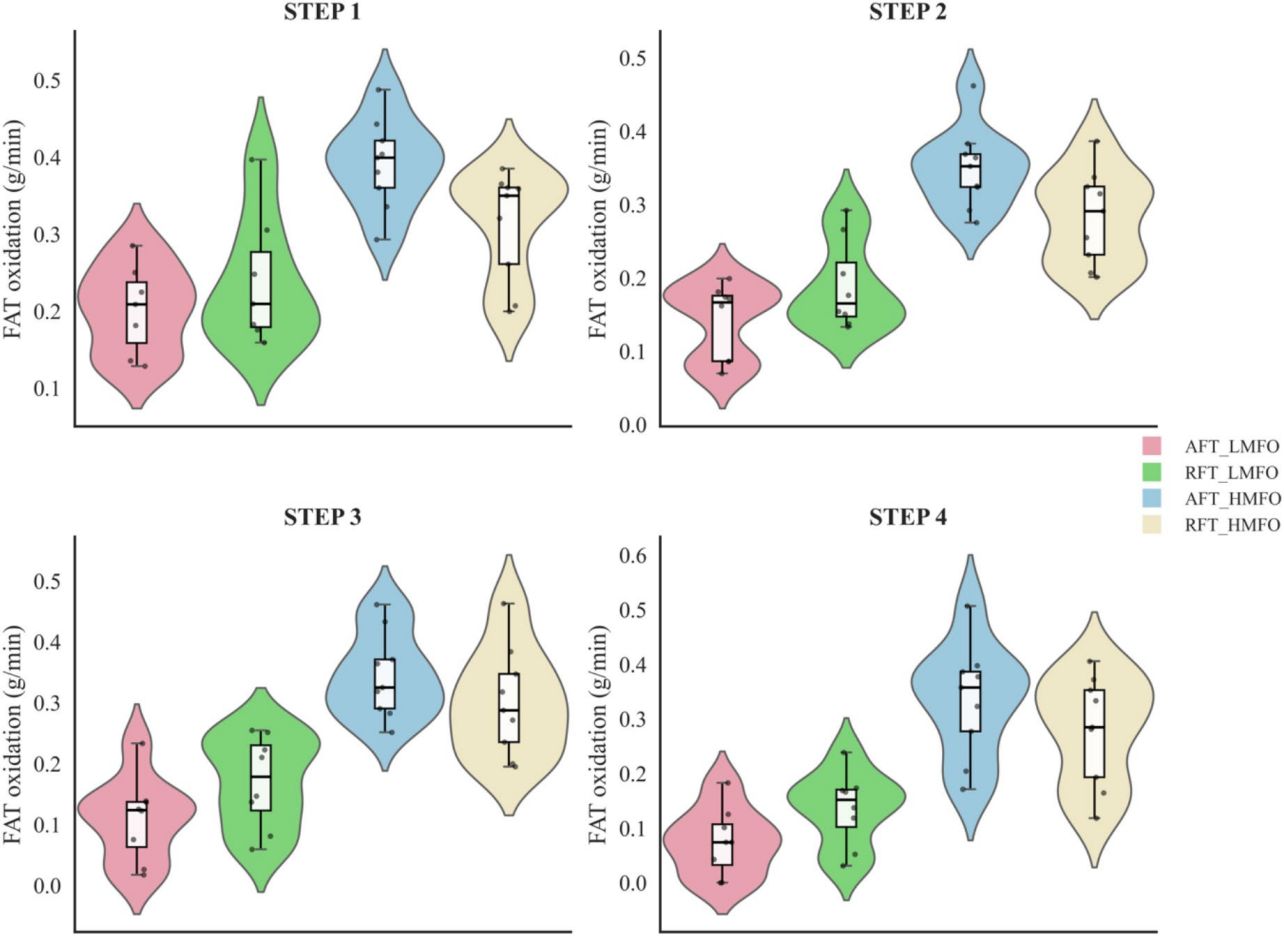
Violin plot of FAT oxidation (g/min) distribution around the first four steps of each test: Absolute FATmax test (AFT) versus Relative to body mass FATmax test (RFT). The internal box plot represents the median and interquartile range, while whiskers indicate the data range. MFO, Maximal Fat Oxidation Point; L‐MFO, low MFO group; H‐MFO, high MFO group.

Figures 2 and 3 illustrate the dispersion between the first four intensities as a function of protocol and group for both RER and FATox. L‐MFO females exhibited larger dispersion, especially in AFT protocol in RER, with more concentrated data within the interquartile range. Contrary, AFT showed lower dispersion and more concentrated data within the interquartile range in H‐MFO.

The violin plots of FATox in Figure 3 exhibited in L‐MFO group a greater dispersion in the RFT protocol. This greater dispersion was maintained in this protocol in H‐MFO, which is increasing dispersion at each step, in this case in both RFT and AFT. Also to note, the coefficients of variation of FATox across these first four intensities (both, for AFT and RFT) exhibited minimal values. Although dispersion remain minimal, RER coefficients in AFT were larger compared to RFT in steps 1 and 4 whatever the group (step 1: 4.12 vs. 3.16% in L‐MFO, 4.74 vs. 3.73% in H‐MFO; step 4: 4.00 vs. 3.57% in L‐MFO, 3.53 vs. 3.12% in H‐MFO despite the group).

### 3.2 Reliability and consistency between FATmax tests

**FIGURE 4:**
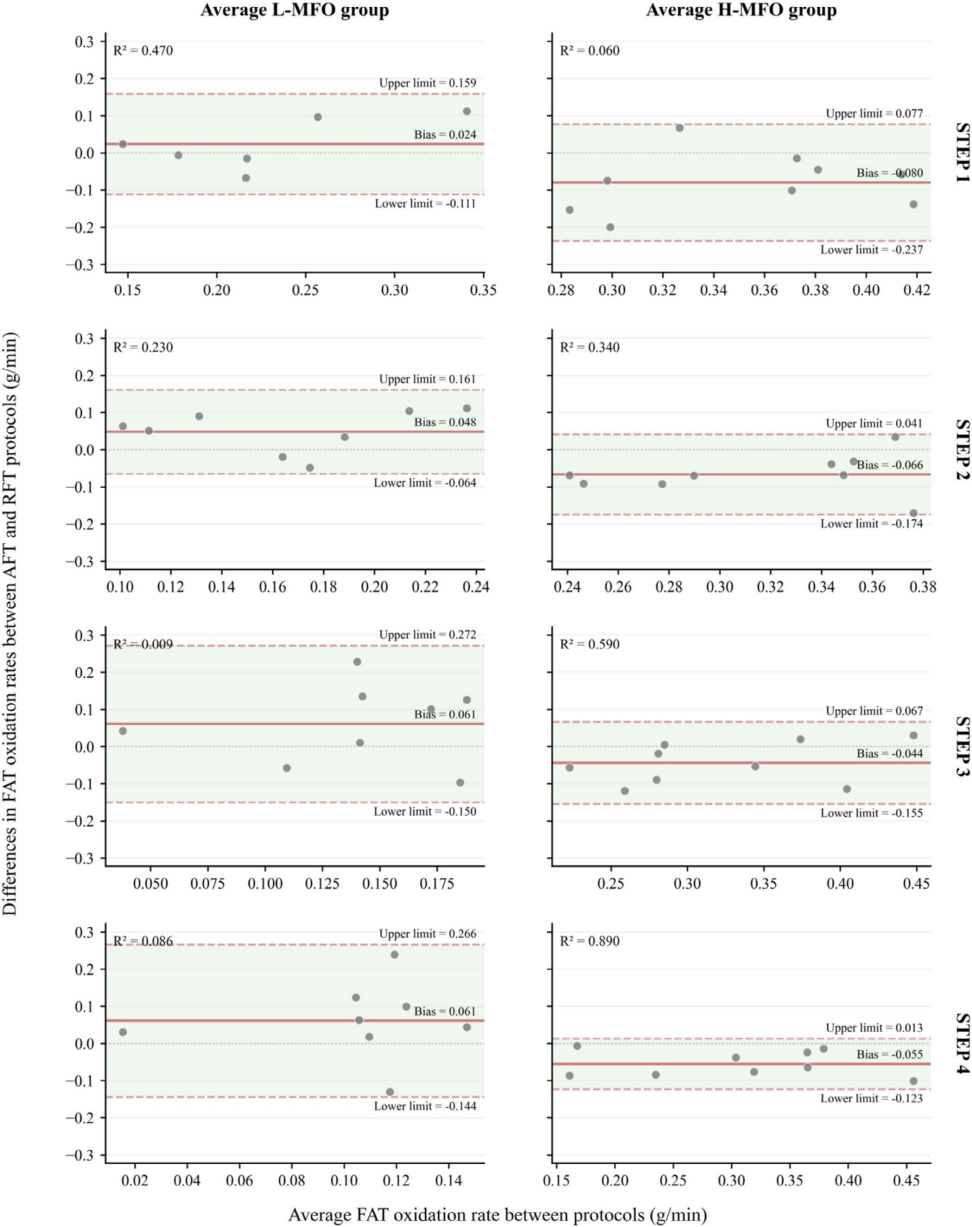
Bland–Altman plots for FATox differences between AFT and RFT. MFO, Maximal Fat Oxidation; L‐MFO, Low MFO group; H‐MFO, High MFO group.

Bland–Altman plots (Figure 4) illustrate the agreement in FATox assesment between RFT and AFT protocols. The limits of agreement (LoA) were relatively narrow, indicating consistency between methods. In the H‐MFO group (right column), the mean bias was low and negative across all steps, with a moderate to large explained variabilities in correlation coefficients (33%, 59% and 89% in steps 2, 3, and 4 respectively). L‐MFO group showed a small but positive mean bias, which increased slightly at each step. Explained variabilities were larger only in steps 1 and 2 (47 and 23%, respectively) with minimal contribution thereafter.

## 4 DISCUSSION

To the best of our knowledge, this is the first study to introduce a FATmax protocol which controls both starting and increasing intensity by power relative to body weight (w/kg) compared with a traditional absolute power protocol. Importantly, the study was not designed to assess protocol reproducibility.

The primary finding is that, although both protocols yielded similar group‐level MFO values and relative power at MFO intensity, the RFT protocol reflected a more consistent progression of fat‐oxidation kinetics and a systematically longer time to FATmax in the L‐MFO group. Longer times in RFT (both time to MFO and step's length) might support a superior suitability of the RFT for detecting true MFO values in individuals with low fat‐oxidation capacity in addition to enhanced analysis of efficiency variables, limited by the attenuated oxidative time zone in this population (Blasco‐Lafarga et al., 2022), or autonomic analysis, which requires longer stable recordings (Shaffer & Ginsberg, 2017). Future studies should determine whether these methodological characteristics improve the validity and determination of FATmax assessment in postmenopausal females and other populations with limited fat oxidation capacity.

As secondary findings, according to the violin plots, the comparison of the first four stages (all with RER <0.90) showed that females in the L‐MFO subgroup consistently exhibited lower RER values, higher FATox rates, and a positive mean bias when RFT was compared with AFT.

These results suggest that the RFT protocol elicits higher fat oxidation responses at lower intensities in individuals with low MFO. In contrast, the AFT protocol was associated with comparatively lower FATox values during the early stages of the test. Interestingly, both subgroups showed very similar descriptive characteristics, with matched age values, despite exhibiting markedly different MFO values. This is consistent with recent evidence indicating that MFO is largely independent of age in postmenopausal females (Blasco‐Lafarga et al., 2022), suggesting that factors other than chronological age likely drive the substantial variability in MFO observed in this cohort.

It should also be acknowledged that resting systolic blood pressure values were elevated across the cohort, despite participants being initially screened as apparently healthy and free from diagnosed cardiovascular disease. Although no between‐group differences were observed, prehypertensive profiles may have contributed to interindividual variability in substrate oxidation responses.

On the one hand, the relative power achieved at MFO showed a whole sample similarity between protocols, and this similarity was retained when the sample was divided into groups. However, although both protocols converged at MFO, their internal progression differed in how workload was distributed across stages. When examining the step‐by‐step behavior of each protocol, some discrepancies emerged as lower relative power for RFT steps 1 and 4, despite producing metabolic and cardiovascular responses similar to AFT at those same stages. Also the observation of a delay equivalent to two incremental steps and thus a longer duration of the fat oxidation predominance compared to the AFT protocol in H‐MFO (i.e., 6 min 30 s more), might lead to a later crossover point in the RFT protocol (Brooks & Mercier, 1994). And to note, this significantly longer duration in RFT for the L‐MFO group (32% time longer) coincided with no statistically significant differences—but a moderate to large effect size—in MFO rate (0.22 g/min in RFT vs. 0.19 g/min in AFT; Cohen's *d* = 0.65), despite lower VO_2_ (12.8 mL/min/kg in RFT vs. 15.4 mL/min/kg in AFT; Cohen's *d* = 0.53). Contrary, females in H‐MFO showed similar values in VO_2_ (17.2 mL/min/kg in RFT vs. 16.5 mL/min/kg in AFT; Cohen's *d* = 0.25). Although the independent contribution of VO_2_ to interindividual variability in fat oxidation capacity cannot be fully determined, these findings suggest that the more gradual progression and prolonged oxidative phase of the RFT may facilitate similar or higher fat oxidation in females without requiring greater oxygen consumption. This methodological characteristic may be particularly relevant in populations characterized by limited oxidative capacity and high interindividual variability in substrate utilization.

The variability in MFO may reflect interindividual differences in factors such as mitochondrial function, capillarisation, and muscle fiber type distribution, as well as physical activity levels in our postmenopausal females. In addition, although mitochondrial content is generally lower in older compared with younger adults, evidence suggests that these declines may be more strongly associated with physical inactivity than with aging per se (Grevendonk et al., 2021).

As hypothesized, our data suggest that the approach may be more sensitive for detecting small differences in fat oxidation in those with low FATox. This may be attributable to the duration of the RFT steps and the more progressive increase in intensity per step, a factor that is especially beneficial to L‐MFO due to a longer period where fat oxidation is predominant. The overall‐sample RFT delay might be advantageous because of the downward and leftward shift in fat oxidation curve, as previously reported (Monferrer‐Marín et al., 2024), meaning that the intensity at which MFO occurs is reached later and at lower workloads. The longer duration of each RFT intensity could lead to higher substrate availability, and a better attainment of steady state, which would be another factor in favor of this protocol. Conversely, the shorter duration of the AFT protocol might limit its ability to fully capture the slower metabolic activation characteristic of individuals with lower cardiorespiratory capacity, underestimating the peak of fat oxidation (Chrzanowski‐Smith et al., 2018).

The sensitivity of muscle relative power (w/kg) to age‐related changes (Alcazar et al., 2020), along with the RFT ability to allow a fairer comparison considering the large heterogeneity in this population (Ireland et al., 2022), suggests that the RFT protocol might be a better approach in this population. The RFT protocol, which normalizes intensity in relation to body weight, provides compensation for interindividual differences in body size and composition within the study population. In this context, RFT may represent a suitable alternative for populations with potentially reduced fat oxidation capacity, enabling a better assessment of substrate utilization across individuals with differing body sizes and capacities. Moreover, the longer duration of this protocol may offer additional benefits when analyzing complementary variables, such as heart rate variability or mechanical efficiency, thereby providing a more comprehensive physiological characterization.

The authors acknowledge some limitations. First, although oxygen uptake was continuously measured and peak VO_2_ values were obtained, lack of VO_2_max outcomes may limit the interpretation of whether interindividual differences in maximal fat oxidation were influenced by differences in maximal aerobic capacity. Second, dichotomizing participants based on a threshold of 0.3 g/min of MFO may oversimplify the physiological continuum of fat oxidation capacity, although this threshold was selected based on previous reference values in comparable populations. Third, the design of the RFT and AFT protocols simultaneously modified two variables (stage duration and increment size), which limits the attribution of the observed effects specifically to the relative scaling of the workload. In addition, workload was scaled relative to body mass rather than fat‐free mass, which may be more closely associated with skeletal muscle oxidative capacity.

Priority was given to a single protocol that would apply these longer stage durations with lower relative workload increments in order to achieve an improved determination of fat oxidation populations with low MFO, such as active postmenopausal females. Future studies should determine whether workload prescription relative to fat‐free mass further improves the FATmax protocols assessment in postmenopausal females.

## 5 CONCLUSION

In summary, data suggest a broad agreement between protocols in the assessment of MFO and FATox rates, with a delay in reaching MFO in the RFT protocol. Although these findings should be interpreted cautiously, the larger fat‐predominant zone of RFT may represent a potential advantage, as it allows a more accurate determination of MFO in a group characterized by high interindividual dispersion in fat‐oxidation capacity and body composition. Longer time to FATmax in the RFT protocol was preserved when the sample was divided into the two MFO subgroups, and this occurred despite both groups showing similar relative power outputs and fat oxidation rates. Further research with larger samples is needed to better understand the responses observed in heterogeneous populations and to optimize FATmax protocols, with the goal of enabling a more accurate and comprehensive characterization of fat oxidation dynamics.

## AUTHOR CONTRIBUTIONS

**Jordi Monferrer‐Marín:** Conceptualization; data curation; formal analysis; investigation; methodology; resources; software; supervision; validation; visualization. **Ainoa Roldán:** Conceptualization; data curation; investigation; methodology; supervision; validation. **Jørn Wulff Helge:** Validation; visualization. **Cristina Blasco‐Lafarga:** Conceptualization; data curation; funding acquisition; investigation; methodology; project administration; resources; supervision; validation; visualization.

## FUNDING INFORMATION

J.M.‐M. was granted funding from a predoctoral contract by the Regional Ministry of Education, Universities and Employment (CIACIF/2022/368; 2023–2027).

## CONFLICT OF INTEREST STATEMENT

The authors declare no conflicts of interest.

## ETHICS STATEMENT

All participants volunteered to participate and provided written informed consent. The protocol was approved by the Science Ethics Committee (2024‐FIS‐3251696) and conducted in accordance with the ethical standards set forth in the Declaration of Helsinki.

## CLINICAL TRIAL REGISTRATION

This study was part of a larger research study investigating associations and changes in Metabolic Flexibility and Autonomic Control After Two Training Programs (Muscle Power vs. Metabolic Power), under the acronym POWER Health, registered as a clinical trial (NCT06336070).

## Data Availability

The datasets generated and/or analyzed during the current study are not publicly available due to the conditions of the ethical approval provided by the Valencia University Human Research Ethics Committee. Notwithstanding, the anonymous data and analysis are available from the corresponding author on reasonable request.
